# Frequency of genetic diseases and health coverage of children requiring admission in a general pediatric clinic of northern Greece

**DOI:** 10.1186/1824-7288-36-9

**Published:** 2010-01-26

**Authors:** Theodoros Lialiaris, Elpis Mantadakis, Dimitra Kareli, Panagiotis Mpountoukas, Aggelos Tsalkidis, Athanassios Chatzimichail

**Affiliations:** 1 Department of Genetics, Democritus University of Thrace Medical School, Dragana, Alexandroupolis, 68100, Greece; 2 Department of Pediatrics, Democritus University of Thrace Medical School, Dragana, Alexandroupolis, 68100, Greece

## Abstract

**Background:**

In order to estimate the causes of pediatric morbidity in our area, with particular emphasis on diseases with a genetic background, we retrospectively categorized the admissions of all children hospitalized in the Department of Pediatrics of the University General Hospital of Alexandroupolis, in the area of Evros, Thrace, Greece over the three year period 2005-2007. Finally, in order to guide health care administrators to improve the delivery of pediatric health care services, we estimated the percentage of hospitalized children who were uninsured and the type of health insurance of those who had medical coverage.

**Patients and Methods:**

The causes of admission, as recorded in the medical records were categorized in terms of the major organ and/or system involved and/or the underlying pathology, with emphasis on diseases with a genetic background. Duplicate admissions, i.e., admissions of the same child for the same underlying disease were excluded. Additional information recorded was age, sex, and type of health insurance of all admitted children. Distribution of the causes of admission by study year was compared by chi-square. A *p *value < 0.05 was considered significant.

**Results:**

Over the study period, there were 4,947 admissions in 2,818 boys and 2,129 girls. Respiratory diseases were the most common accounting for 30%, while infectious diseases followed with 26.4%. The frequency of chromosomal abnormalities among the hospitalized children was only 0.06%. However, if we consider diseases with an underlying genetic background, this percentage rises to 5%. Approximately 10.3% of the admitted children had no health insurance.

**Conclusions:**

The percentage of children hospitalized in our area due to a disease with an underlying genetic background was 5%. This percentage pertains to a Department of Pediatrics that has no inpatient subspecialty units and which is located within a General hospital, because hospitalizations for genetic diseases are more frequent in specialized pediatric hospitals, with competence in clinical genetics. The double figure of uninsured children is worrisome and dictates the need for governmental efforts for universal pediatric health coverage in our country.

## Background

In the last 40 years, several studies investigated the frequency of genetic diseases in pediatric hospitals in developed countries [1-5]. However, to the best of our knowledge, such a study has never been done in Greece. The University General Hospital of Alexandroupolis is the teaching hospital of the Medical School of the Democritus University of Thrace. It is located in the area of Evros, Thrace, in Northern Greece, and has a well-organized and adequately staffed Department of Pediatrics with 40 beds. However, no inpatient subspecialty units (e.g., Hematology/Oncology, Nephrology, ICU, etc.) exist within this Department.

The purpose of the current work was to categorize the admissions in the Department of Pediatrics of the University General Hospital of Alexandroupolis over the period 2005-2007 by the main system/organ involved, to calculate the frequency of diseases with a genetic background in the hospitalized children, and finally to estimate the percentage of hospitalized children who were uninsured, and the type of health insurance of those who had medical coverage. Although the percentage of genetic diseases and the availability of health coverage in hospitalized children are not directly linked, knowledge of both is useful for planning better pediatric services in our area.

## Patients and Methods

We hand-searched the medical records of the Department of Pediatrics of the University General Hospital of Alexandroupolis for all children who were hospitalized over the three-year period 1/1/2005-31/12/2007. Our hospital which is a General University Hospital has a system of recording electronically the name of the child admitted to the Department of Pediatrics along with the final diagnosis. Since this process is not free of risks for mistakes, we hand-searched the medical records of all admitted children, not just the discharge summaries, in order to be confident that the correct diagnosis was recorded. Additional information extracted from the medical records was sex, and type of health insurance of the admitted children. Although data extraction from the medical records was done by personnel without a medical degree (DK, PM), assignment to the categories of diseases with a genetic background was supervised by the first two authors who are physicians (TL and EM). Duplicate admissions, i.e., admissions of the same child for the same underlying disease were excluded.

Diseases were categorized in terms of the major organ and/or system involved and/or the underlying pathology, except in cases of unspecified organ and/or system involvement, when inclusion into the others category was assigned. Respiratory diseases of known infectious etiology (e.g., pneumonias with positive blood cultures for *Streptococcus pneumoniae*) were grouped as infectious diseases. Infectious diseases of the respiratory tract of unknown etiology were grouped under the respiratory diseases category.

In case that a child with a known single-gene or chromosomal syndrome was admitted for a common pediatric problem (e.g., a febrile respiratory infection), both diagnoses i.e., the syndrome and the infection were recorded. This created few duplicate entries that however, did not alter our overall results.

We used a categorization of diseases similar to that of Shawn et al., which is a modification of an earlier work by Hall et al, in order to allow for the increased recognition of the genetic contribution of various chronic medical conditions [1,3]. Table 1 describes the categories used, as well as examples from each category. In particular, category I (*underlying conditions with strong genetic basis*) includes individuals with congenital underlying conditions, with various subcategories designed to further refine the genetic component. Category II (*birth defects without known genetic basis*) includes individuals with birth defects that are not known to have a genetic basis, including teratogenic effects. This classification does not imply that possible genetic causes have been disproved, but only that current evidence is not strong enough for an underlying genetic cause to be considered in their pathogenesis. Category III (*acquired disorders with genetic predisposition*) includes individuals with acquired disorders for which the medical literature and clinical experience suggest a genetic component, predisposition, or genetically determined susceptibility [3].

**Table 1:** Determination of disease categories with genetic background in the hospitalized children along with clinical examples.

Category	Definition	Examples
1	**Underlying conditions with strong genetic basis**	
*1A*	*Single-gene or chromosomal*	Hemophilia, thalassemia, ataxia telangiectasia, Down's syndrome, cystic fibrosis
*1B*	*Multifactorial/polygenic*	Hydrocephaly, congenital hydronephrosis
*1C*	*Heterogeneous causes, often of a genetic basis*	Psychomotor retardation, megalocephaly, microcephaly, mental retardation
2	**Birth defects without known genetic basis**	
*2A*	*Malformation of unknown etiology*	-
*2B*	*Teratogenic disorders*	-
3	**Acquired disorders with genetic predisposition**	Diabetes mellitus, asthma

All admissions were categorized into four age groups: <1 month (neonates), 1 month-1 year (infants), 1 year-5 years (pre-school children), and >5 years. Possible sex differences between children who suffered from diseases with a genetic background compared with those who did not suffer from such a disease and distribution of the causes of admission by study year was compared by chi-square. A *p *value < 0.05 was considered significant.

## Results

There were 5,395 admissions over the study period, of which 448 were excluded because they were duplicates, leaving a total of 4,947 eligible admissions. Approximately 30% of the admissions were due to respiratory diseases, with no significant change over time. The second most common category included infectious and parasitic diseases (26.4%), followed by genitourinary (6%), neurologic (5.8%), and gastrointestinal diseases (4.4%), poisonings and accidents (3.9%), blood diseases (2%), circulatory system diseases and endocrine and metabolic diseases (with 0.8% each), cutaneous diseases (0.7%), musculoskeletal diseases (0.6%), collagen vascular diseases (0.5%), behavioral disorders (0.4%) and chromosomal abnormalities and syndromes (0.06%). Approximately 17.6% of the admissions belonged to the "others" category. There was a statistically significant decrease over time in neurologic, blood and circulatory system diseases (*p *< 0.05). Among the 4,947 admissions, 246 (approximately 5%) were due to diseases with an underlying genetic background. Detailed information for these admissions including sex and age of the admitted children are given in Table 2. Table 3 shows the categorization of admissions by major system involved and/or underlying etiology by study year. Overall, 57% of the admitted children were boys. Among children suffering from a disease with a genetic background, approximately 62% were males. There was no significant sex difference between children who suffered from diseases with a genetic background compared with those who didn't. Most children (42%) requiring hospitalization were 1-5 years old, followed by children aged >5 years, infants, and neonates with 32%, 25% και 1%, respectively.

**Table 2:** Information for the number of admitted children, their sex and age based on the categories with genetic background of Table 1.

Category	Definition	No of patients	Females	Males	<1 m*	1-12 m	1-5 y*	>5 y
1	**Underlying conditions with strong genetic basis**							
*1A*	*Single-gene or chromosomal*	22 (8.9%)	5	17	-	6	9	7
*1B*	*Multifactorial/polygenic*	10 (4%)	2	8	1	2	4	3
*1C*	*Heterogeneous causes, often of a genetic basis*	20 (8.1%)	7	13	1	5	8	6
2	**Birth defects without known genetic basis**							
*2A*	*Malformation of unknown etiology*	-	-	-	-	-	-	-
*2B*	*Teratogenic disorders*	-	-	-	-	-	-	-
3	**Acquired disorders with genetic predisposition**	194 (79%)	78	116	3	48	81	62
	**TOTAL**	246	92	154	5	61	102	78

m: month, y: year

**Table 3:** Categorization of admissions by major system involved and/or underlying etiology.

		2005	2006	2007	TOTAL
1	Respiratory diseases	525	513	446	1484 (30%)
2	Circulatory system diseases	14	21	6	41 (0.8%)
3	Blood diseases	51	25	24	100 (2%)
4	Genitourinary tract diseases	115	99	86	300 (6%)
5	Gastrointestinal diseases	64	74	77	215 (4.4%)
6	Neurological diseases	116	107	65	288 (5.8%)
7	Collagen vascular diseases	11	7	5	23 (0.5%)
8	Musculoskeletal diseases	12	12	5	29 (0.6%)
9	Cutaneous diseases	14	15	5	34 (0.7%)
10	Endocrine and metabolic diseases	12	13	13	38 (0.8%)
11	Infectious diseases	431	464	412	1307(24.6%)
12	Behavioral disorders	9	4	3	16 (0.4%)
13	Chromosomal abnormalities and syndromes	0	3	0	3 (0.06%)
14	Poisonings - Accidents	75	71	50	196 (3.9%)
15	Others	300	285	288	873 (17.6%)
	**TOTAL**	**1749**	**1713**	**1485**	**4947**

In each of the three study years, most children (39.4%) were insured by the foundation of social insurance (IKA) that is responsible for the health coverage of people working in the private sector of the economy. A total of 17.4% of the children were insured by the national government, because one or both parents worked in public positions, another 13.7% were insured by the publicly-funded agricultural insurance agency, 10.5% of very low-income patients had welfare and 6.7% were insured by the fund for merchants and self-employed small-business owners. Children insured with minor health insurance agencies were grouped together and constituted 1.6% of the total admissions. Remarkably, 10.3% of the admitted children were uninsured.

## Discussion

The goals of this retrospective study were to categorize by system and/or organ involved the admissions in the Department of Pediatrics of the University General Hospital of Alexandroupolis over a three-year period, to calculate the documented frequency of genetic diseases and of diseases with a genetic background in the hospitalized children, and finally to estimate the percentage of hospitalised children who were uninsured and the type of health insurance of those with medical coverage.

Approximately 56% of the admissions were due to respiratory and/or infectious problems that are usually more prominent during the winter months. Although only 0.06% of the admitted children had a known chromosomal abnormality, if we consider diseases with a genetic basis, this percentage rises to 5%. This figure, although lower than the double figure of admissions associated with environmental factors (i.e., respiratory and/or infectious problems), demonstrates that genetic and "partially" genetic diseases impose a considerable financial burden in the delivery of pediatric health services in our area. Moreover, this percentage is in all likelihood an underestimate of the true impact of genetic diseases in pediatric inpatient care, since as previously explained our Department cares only for children with general pediatric problems and has no specialized subspecialty inpatient units.

Review of the medical literature reveals that the percentage of children with a genetic disease or a disease with an underlying genetic background is substantial and requires our attention. For example, according to the 2006 annual review of vital statistics in USA, congenital malformations, deformations, and chromosomal abnormalities accounted for 4.6% of all deaths in the age group 1 to 19 years[6]. More recent studies show that 98% of children admitted over a year in a children's hospital had indications for illnesses with a genetic etiology, even though this was not demonstrated on the final diagnosis and release papers [3]. Likewise, Hall et al. reported that in a general pediatric hospital, 4.5% of admitted children had a clear genetic disorder, and that 48.9% of admissions were due to a multi-factorial/polygenic disorder (22.1%), developmental anomaly (13.6%) or familial disorder (13.2%). Although our categorization is a modification of Hall et al, it is worth mentioning that in our Department of Pediatrics that is located within a General Hospital 8.9% of admitted children had a single-gene or chromosomal disease (category 1A), 4% had multifactorial/polygenic (category 1B) diseases, 8.1% had heterogeneous causes for admission, often of a genetic basis (category 1C) and finally 79% had acquired disorders with genetic predisposition (category 3) (Table 2).

At this point, we should mention that the contribution of genetic disorders and congenital malformations in the total number of admissions is much higher in pediatric long-term care facilities, and specialized pediatric hospitals, in particular those with competence in clinical genetics. For example, a study that was performed in a pediatric long-term care and rehabilitation center revealed that genetic disorders and congenital anomalies were responsible for 50% of the overall admissions and for 60% of end-of-life care admissions [7]. Furthermore, a considerable percentage/number of genetic disorders is not diagnosed during the early years of life. Thus, the management of genetic diseases imposes substantial societal, medical and financial burdens in the delivery of pediatric health services, albeit smaller than that of diseases with environmental pathogenesis.

Substantially more boys than girls required hospitalization in our department. This finding is in agreement with the available literature showing that boys at a young age appear to be more susceptible, especially to infections than girls [1,5]. Among admitted children suffering from a disease with a genetic background, approximately 60% were males, i.e., the percentage of males with a genetic disease was proportional to the percentage of admitted boys (57%). Finally, irrespective of sex and underlying disease, most children (42%) requiring hospitalization were 1 to 5 years old. Regarding the significant decrease in the number of neurologic, blood and circulatory system diseases over time, we have no explanation for this finding, although our study was only extended over a three-year period and longer follow-up over a decade or more may prove this finding to be due to chance.

Regarding health coverage, as expected, most hospitalized children were covered under IKA, an organization that insures workers in the private sector of the economy. These workers are allowed to visit a limited number of pediatricians, who are paid a salary irrespective of the number of insured children examined. Hence, the parents need to pay money out of pocket in case they prefer to have their child examined by a private pediatrician who has no contract with IKA. Moreover, IKA does not operate facilities for inpatient care. That is why the parents of children who are insured with IKA frequently prefer to visit hospitals of the National Health System, like our hospital, for free medical services, especially if there is a possibility their child will require hospitalization.

A very interesting and worrisome finding of this study is that 1 in 10 children requiring admission in our hospital is uninsured and another 10.5% originates from families with extremely low income that are on welfare. Hence, despite of the presence of a National Health System in Greece, many children remain completely uninsured or have limited health coverage (welfare). We consider that these figures are worrisome and indicative of the relatively poor socioeconomic standards and living conditions of our area that has many immigrants and people with different educational, religious, and social backgrounds. Consequently, we believe that the Greek government should increase its efforts for providing universal pediatric health coverage, an achievable and not idealistic goal, something that may not be the case for adults due to the much higher cost required.

In conclusion, the percentage of children with a genetically determined disease and with partially genetic diseases requiring admission in a Department of Pediatrics located within a general hospital of Northern Greece is approximately 5%, a figure that is in close agreement with the available medical literature that originates from general not specialized pediatric hospitals. Our results can assist health-care administrators to plan for the health needs of the children in our area, and moreover justify the development and funding of a specialized, academic department for the study of genetic diseases that in close cooperation with the Department of Pediatrics will provide quality services for the children of our area [4,7-9].

## Competing interests

The authors declare that they have no competing interests.

## Authors' contributions

TL designed the study, supervised the assignment to each admission category, and drafted the manuscript. EM supervised the assignment to each admission category, performed the statistical analysis, and made major revisions in the submitted manuscript. DK and PM extracted data from the medical records. AT and AC participated in the designing the study. All authors read and approved the final manuscript.

## Ethical approval

The authors declare that no ethical approval was required for this study.
